# Long‐term follow‐up after regional nodal irradiation: What wisdom comes with age

**DOI:** 10.3322/caac.70089

**Published:** 2026-05-16

**Authors:** Youssef H. Zeidan, Stephen A. Mihalcik, Rachel B. Jimenez

**Affiliations:** ^1^ Department of Radiation Oncology Baptist Health South Florida Lynn Cancer Institute Boca Raton Florida USA; ^2^ Department of Radiation Oncology Massachusetts General Brigham Cancer Institute Boston Massachusetts USA

In 2015, two seminal randomized clinical trials evaluating the utility of regional nodal irradiation (RNI)—National Clinical Trials of Canada trial MA.20 and European Organization for Research and Treatment of Cancer (EORTC) trial 22922 (ClinicalTrials.gov identifiers NCT00005957 and NCT00002851, respectively)—were published simultaneously with similar end points and findings. At a median follow‐up of 10 years, neither study demonstrated a significant difference in their shared primary end point of overall survival (OS). However, both reported improvements in disease‐free survival (DFS), distant DFS, and locoregional disease control favoring RNI.^1^, ^2^

Kaidar‐Person and colleagues now report the 20‐year results of the EORTC 22922 trial, which enrolled patients between 1996 and 2004, in *CA: A Cancer Journal of Clinicians*.^3^ To recall, this trial enrolled women with both node‐negative and central or medially located tumors (45%) and those with axillary nodal involvement (55%). At 20 years, no significant differences were observed in OS with the addition of RNI. It is important to note that the previously reported improvements in DFS and distant DFS with internal mammary–medial supraclavicular (IM‐MS) irradiation at 10 years were no longer evident at 15 years, and this remained the case at 20 years. In contrast, improved breast cancer mortality was sustained at 20 years with IM‐MS, representing a nearly 20% relative reduction in breast cancer mortality among patients receiving RNI. However, in contrast to the previously published 10‐year and 15‐year outcomes, a significant increase in non‐breast cancer–related deaths in the IM‐MS arm was observed at 20 years of follow‐up.

With a study of this size (>4000 patients) and scope, the authors are to be commended for adhering to the planned trial analysis timeline and for reporting final outcomes at 20 years. The increasing incidence of breast cancer, the alarming rise in cases among young women, and the growing number of aging survivors all underscore the importance of having such long‐term data.^4^ In fact, this is one of only a handful of randomized trials reporting radiation therapy outcomes at 20 years, with well described quality‐assurance and follow‐up patterns.^5^, ^6^

Although this publication represents an impressive and laudable accomplishment on behalf of the entire study team, appropriate interpretation of these long‐term outcomes is necessarily complicated by the limitations that arise with time. During the trial's enrollment period and in the years since, breast cancer detection and treatment have evolved dramatically. For example, whereas the study participants were not characterized with respect to human epidermal growth factor receptor 2 (HER2) status, HER2 assessment has since become standardized and mandated. The recurrence score has been incorporated into staging and treatment algorithms for those with estrogen receptor‐positive disease. Specific chemotherapy regimens were not required on the trial whereas, in modern practice, we eschew fluorouracil in favor of taxanes, use dose‐dense administration, incorporate HER2‐targeted and immunotherapeutic agents, and use adjuvant cyclin‐dependent kinase 4/6 and polymeric (adenosine diphosphate‐ribose) polymerase inhibitors to more effectively target the biology of individual cancer types. In today's practice, a substantial portion of the enrollees would likely also receive neoadjuvant therapy. One can imagine that the development of more effective and targeted systemic regimens could affect the study outcome in both positive and negative ways: a diminished risk of distant recurrence may increase the value of RNI, as suggested by the 2023 Early Breast Cancer Trialists’ Collaborative Group meta‐analysis,^7^ whereas improvements in neoadjuvant systemic therapy efficacy beyond that threshold may diminish its value by supplanting its role in locoregional control as observed in National Surgical and Bowel Project trial B‐51 (ClinicalTrials.gov identifier NCT10182975).^8^

Similarly, the surgical approach to the axilla has undergone significant refinements. Patients enrolled on EORTC 22922 received either mastectomy or lumpectomy with axillary lymph node dissection. In a similar cohort receiving treatment today (i.e., 44% node‐negative, 43% with from one to three involved axillary nodes), a majority would likely proceed with sentinel lymph node biopsy alone or, in select cases, omit axillary surgery altogether.^9^, ^10^, ^11^, ^12^, ^13^ With less reliance on surgery for regional control, adjuvant radiation may offer relatively greater value.

Finally, given the concern that radiation late effects may outweigh the benefits observed with RNI at long‐term follow‐up, the authors rightly note that the evolution of radiation techniques is also limiting. Since the trial enrollment period, radiation design has moved firmly from a two‐dimensional to a three‐dimensional era, with techniques that reflect a higher priority on cardiac‐sparing, judicious deployment of image guidance, deep inspiration breath‐hold techniques, and intensity‐modulated radiation therapy. Collectively, these advances allow for a drastic reduction in mean heart doses from those reported on the trial (18.3 Gray in the RNI group) to a more common acceptable limit of <3 Gray.^14^

The authors acknowledge that limitations in data collection prevent more complete characterization of cardiovascular toxicities and causes of death, which further confound the interpretation. Although radiation‐related cardiac toxicity is proposed as a rational causal link from the study intervention to the newly observed increase in non‐breast cancer deaths, there are no data with which to establish the cause of this finding. The authors posit that this could alternatively be attributed to the impact of other late radiation effects or perhaps an increased rate of competing risks that the patients in the RNI arm have survived long enough to experience.

Therein lies both the privilege and the consternation of interpreting and applying long‐term clinical trial outcomes. If this study were performed with modern cardiac‐sparing techniques, would we observe the same decrement with respect to non‐cancer deaths at 20 years? Most clinical trials, owing to financial and practical implications, routinely complete follow‐up at 10 years. What potential late effects would we observe from other cancer interventions if only we kept looking? Is it even necessary? If the benefits to breast cancer mortality and local regional control persist for 20 years after the intervention, should a difference in non‐cancer deaths that first emerges from the data after 20 years practically influence our recommendations?

Although the data continue to raise questions even decades later, they also offer a persistent, clear, and powerful answer: RNI has durable benefits for breast cancer–specific mortality and disease control. Questions remain regarding the appropriate application of RNI in modern practice; however, with approximately one half of all patients in the trial still alive at 20 years' follow‐up, there is no doubt that the long‐term results are relevant to our patients. In the context of modern cardiac‐sparing radiotherapy techniques, clinicians can be assured that non‐breast cancer deaths are unlikely to be recapitulated in modern practice.

## CONFLICT OF INTEREST STATEMENT

Rachel B. Jimenez reports personal/consulting fees from Alexion Pharmaceuticals and Daiichi‐Sankyo Company outside the submitted work. Youssef H. Zeidan and Stephen A. Mihalcik disclosed no conflicts of interest.
